# Acute non-traumatic pancreatitis in a patient with pancreas divisum: a case report

**Published:** 2013-09-25

**Authors:** D Anyfantakis, N Partalis, G Polimili, S Kastanakis

**Affiliations:** * Primary Health Care Centre of Kissamos, Chania, Crete, Greece; ** First Department of Internal Medicine, Saint George General Hospital of Chania, Crete, Greece

**Keywords:** Pancreas divisum, acute pancreatitis

## Abstract

Pancreas divisum is a frequent congenital anatomical anomaly characterized by the failure of fusion of the ducts of Santorini and Wirsung during fetal development. Although the condition usually remains asymptomatic, it has been reported to be a predisposing factor of chronic and recurrent idiopathic pancreatitis. We report a case of acute non-traumatic pancreatitis in a 54-year-old Caucasian male with pancreas divisum. Diagnosis was established based on the findings from magnetic resonance imaging and magnetic resonance cholangiopancreatography. The patient was managed conservatively and was discharged home having an uneventful clinical course after five days of hospitalization.

Although the role of the pancreas in the induction of acute pancreatitis is still a matter of debate, physicians have to be aware about this prevalent pancreatic anatomic abnormality. Timely detection may help in the prevention of potential recurrent pancreatic reaction.

## Introduction

Pancreas divisum, the most common congenital variation of the pancreatic ductal anatomy, occurs in approximately 10% of the population [**1**]. The entity has been positively associated with the induction of idiopathic chronic or recurrent pancreatitis [**2**]. Here we report a case of acute pancreatitis in a previously healthy male.

## Case presentation 

A 54-year-old Caucasian male was referred by his general practitioner to the emergency department of the Saint George General Hospital of Chania, Crete because of a 10-hour history of intermittent abdominal pain located at the epigastric region without radiation accompanied by nausea. His past medical history was unremarkable. There was no previous history of chololithiasis and hypercalcemia. He reported moderate alcohol consumption (one glass of red wine daily). Abdominal examination disclosed marked sensitivity on palpation of epigastric area and of right upper quadrant. His vital signs on admission were as it follows: temperature, 36.8 grade Celsius; blood pressure, 150/90 mmHg; heart rate, 76 beats/min; oxygen saturation, 98% while he was breathing ambient air. The electrocardiogram revealed sinus rhythm. 

 Initial laboratory work up showed: white blood count, 10.72 K/μl (normal range: 4-11 K/μl); hematocrit, 42% (normal range: 40-50%); haemoglobin 14.4 g/dl (normal range: 13.5-17.5 g/dl); platelet counts, 267 μl (normal range: 150-450 K/μl); Renal and liver function tests were all normal. Cardiac enzymes, cholestatic indexes (total bilirubin, direct bilirubin), calcium and C-reactive protein levels were all within normal limits. Pancreatic enzymes were found significantly elevated with an amylase level of 1398 U/L (normal range: 28-100) and serum lipase level of 2543 U/l (normal range: 13-60). An abnormal value of amylase in urine was also recorded [12311 (normal range: 0-460)].

**Fig. 1:**
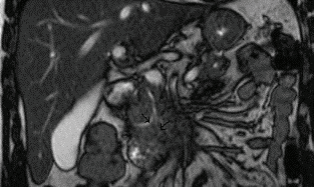
MRI image showing the presence of dorsal pancreatic duct (right) and ventral pancreatic duct (left) that drains into the major papilla

**Fig. 2:**
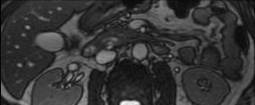
Transverse MRI image, showing the dorsal pancreatic duct the ventral pancreatic duct and the common hepatic duct

 Abdominal ultrasound imaging was normal except for the dilation of the pancreatic duct (d=2.5 cm). A presumed diagnosis of acute pancreatitis was made probably due to a relative outflow obstruction on the pancreatic duct. The patient underwent Magnetic Resonance Imaging (MRI) of the upper abdomen. MRI showed a pancreatic duct abnormality by illustrating two separate pancreatic ducts (dorsal and ventral) raising the diagnostic suspicion of pancreas divisum (**Fig. 1,2**).

**Fig. 3:**
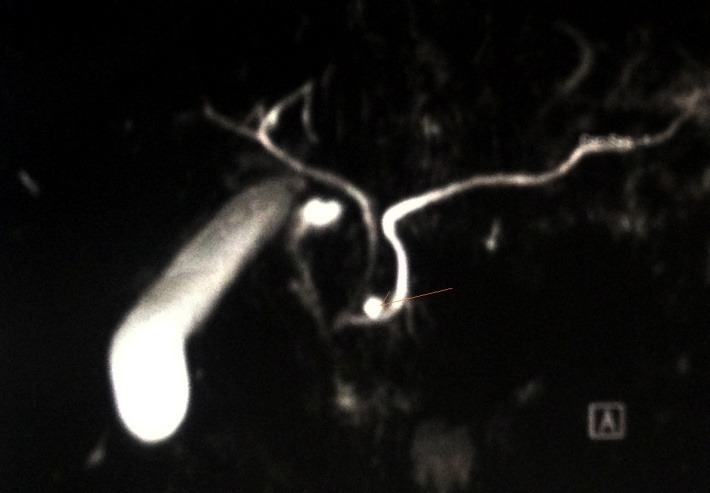
Intersection of the major pancreatic duct with accessory pancreatic duct (arrow)

Further investigation with magnetic resonance cholangiopancreatography (MRCP) revealed findings suggestive of the presence of pancreas divisum (**Fig. 3,6**).

**Fig. 4:**
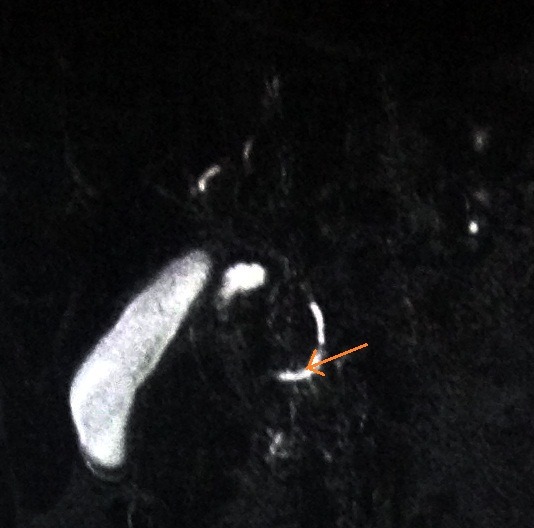
Visualization of the major pancreatic duct (arrow)

**Fig. 5:**
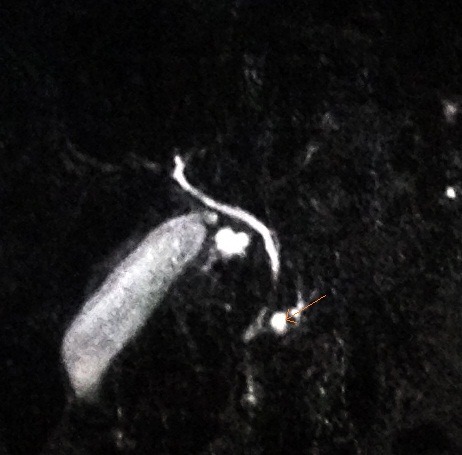
Accessory pancreatic duct is visualized near the major pancreatic duct (arrow)

**Fig. 6:**
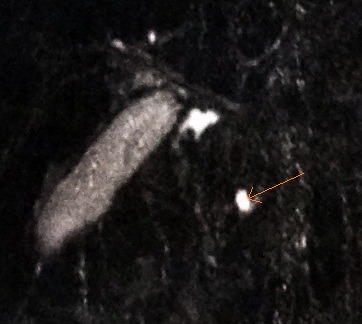
Visualization of the accessory pancreatic duct that follows a different course from major pancreatic duct (arrow)

 The patient received pharmacological treatment with cefoxitin and esomeprazole with significant improvement of his clinical condition after 3 days. He was discharged home on the fifth hospital day receiving dietary advices of alcohol abstinence and avoidance of fatty aliments consumption due to the high risk of recurrent pancreatitis. On follow up examination after 1 month, the patient remained healthy, asymptomatic with normalization of the pancreatic enzymes levels.

## Discussion

We present this case as an argument for the periodical check up of the blood count during the treatment with antithyroid drugs in order to detect anomalies as leukopenia and even worse agranulocytosis. It is difficult to express any certain correlation between the autoimmune thyroid disease as Graves’s disease and the risk of hematological disturbances. It is possible that the patients who develop agranulocytosis actually have more severe marrow diseases as malignancy and the drop of granulocytes is in fact the exposed part of an iceberg. Further serial investigations should be performed. 

 Formation of pancreas occurs during embryonic development by the fusion of the ventral and dorsal pancreatic primordia [**3**]. Fusion of the dorsal and ventral pancreatic ducts forms the major pancreatic duct (Wirsung’ s duct) [**3**] which at rest has a maximum diameter of 2 mm and is responsible for the drainage of pancreatic secretions derived from cap, body and tail of the gland at the major duodenal papilla [**4**]. The accessory pancreatic duct (Santorini’s duct) crosses major pancreatic duct and stops on the minor papilla [**4**]. Minor papilla is located 10-15 cm above the major papilla on the median wall of the 2nd part of the duodenum [**4**].

 A fusion failure of the ventral and dorsal pancreatic buds occurs in pancreas divisum during the 6th to 8th week of fetal development [**3**] resulting in two ‘independent glands’ with two separate ducts [**5**]. The dorsal duct drains the main portion of the pancreas into the minor papilla and the ventral duct drains only a small segment of the pancreas (inferior portion of the pancreatic cap) at the major duodenal papilla [**4**]. Historically, the condition was first described by Joseph Hyrtl (1810-1894) [**6**]. Two types of pancreas divisum are described: complete pancreas divisum, which occurs more frequently with total failure of fusion and the incomplete form where the dorsal and ventral pancreatic ducts are joined through small caliber communicating branch ducts [**4**]. 

 The entity is usually asymptomatic [**3**]. However, insufficient drainage of the exocrine pancreatic secretions may result to high intraductal pressure in the duct of Santorini and induce chronic pancreatitis or recurrent episodes of abdominal pain [**3**]. Almost 2 out of 10 patients with unexplained idiopathic pancreatitis were diagnosed with pancreas divisum [**7**]. Endoscopic retrograde cholangiopancreatography (ERCP) represents the first choice investigation for a definite diagnosis [**3**]. The diagnosis of PD is based on ERCP findings, which include the visualization of a short isolated ventral duct or identification of two separate pancreatic ducts [**8**]. Magnetic resonance cholangiopancreatography (MRCP) is a useful non-invasive technique with high sensitivity and specificity for the diagnosis of pancreas divisum [**3,8**] by showing the intersection of the distal common bile duct with the dorsal pancreatic duct. Transabdominal ultrasonography may be used for the determination of the pancreatic duct diameter [**9**]. A pancreatic duct diameter of more than 1.5 mm for more than 30 minutes following a fatty meal or after secretin infusion is considered abnormal [**9,10**]. Pancreatic ducts can be depicted without the injection of iodinated contrast material [**3**]. Non-communication of the dorsal and ventral pancreatic ducts and the dominance of the dorsal pancreatic duct is visualized [**3**]. Decompression of the Wirsung’s duct, through sphincterotomy of the minor duodenal papilla represents the basic therapeutic modality for cases with recurrent episodes of pancreatitis [**11**]. 

 In our case, pancreas divisum was the only predisposing factor for acute pancreatitis. Although pancreas divisum seems to predispose to chronic and recurrent idiopatic pancreatitis [**2**], its contribution to the induction of acute pancreatitis is still debatable [**2**]. A high level of physicians’ awareness is required for this common pancreatic duct anomaly since timely detection may help prevention of recurrent pancreatitis by encouraging patients to follow a healthier dietetic regimen and avoid alcohol consumption. 

 Consent

 Written informed consent was obtained from the patient for publication of this case report and any accompanying images. A copy of the written consent is available for review by the Editor-in-Chief of this journal. 

 Competing interests

 The authors declare that they have no competing interests.
